# Fifteen Dimensions of Health among Community-Dwelling Older Singaporeans

**DOI:** 10.1155/2011/128581

**Published:** 2011-10-30

**Authors:** Chetna Malhotra, Angelique Chan, Rahul Malhotra, Truls Østbye

**Affiliations:** ^1^Program in Health Services and Systems Research, Duke-National University of Singapore Graduate Medical School, 8 College Road, Level 4, Singapore 169857; ^2^Department of Sociology, National University of Singapore, Singapore 117570; ^3^Department of Community and Family Medicine, Duke University, Durham, NC 27710, USA

## Abstract

This paper aims to present a broad perspective of health of older Singaporeans spanning 15 health dimensions and study the association between self-rated health (SRH) and other health dimensions. Using data from a survey of 5000 Singaporeans (≥60 years), SRH and health in 14 other dimensions were assessed. Generalized logit model was used to assess contribution of these 14 dimensions to positive and negative SRH, compared to average SRH. About 86% reported their health to be average or higher. Prevalence of positive SRH and “health” in most other dimensions was lower in older age groups. Positive and negative SRH were associated with mobility, hearing, vision, major physical illness, pain, personal mastery, depressive symptoms, and perceived financial adequacy. The findings show that a majority of older Singaporeans report themselves as healthy overall and in a wide range of health dimensions.

## 1. Introduction

Over the last 40 years, Singapore, a small island country with a population of about 5 million [1], has made a rapid transition from a developing to a developed economy, with concurrent improvements in civic and health infrastructure. Life expectancy at birth increased from 72 years in 1980 to 80.9 years in 2008 [2], chiefly owing to reduction in mortality due to communicable diseases [3]. Today, Singapore is one of the most rapidly aging countries in Asia. The proportion of older adults (aged 65 years and over) in the population, currently about 8%, is projected to increase to 19% by 2030 [4]. With increase in the proportion of older adults in Singapore and in their life expectancy, their health has become a priority. 

 Data from several countries shows that older adults are spending larger proportions of their lifetimes free from illness and disability [5–9]. Thus, traditional health indicators that measure morbidity in terms of physical health status alone and of mortality are inadequate yardsticks to evaluate the health of older adults. A broader perspective of health is increasingly commonplace, stemming from the World Health Organization's (WHO) definition, “Health is not simply absence of disease or infirmity, but is the presence of a complete physical, mental and social well-being” [10]. The related constructs of successful and healthy aging, though ill-defined, are also considered to consist of multiple dimensions [11–13]. 

A key dimension of health widely used by researchers to gauge the overall health status of older adults is self-rated health (SRH). Several studies have assessed SRH in surveys using a single question asking the respondents to rate their overall health status. This single question on SRH has been found to be an indicator of current health status and predicts future health outcomes and mortality, though the association varies by gender, race, and socioeconomic status [14–20]. Several studies globally have shown that SRH incorporates multiple health dimensions including those representing physical health (chronic diseases, functional limitations, hearing, vision, pain, chewing ability, restful sleep), psychological well-being (depressive symptoms, mastery), and social integration [20–34]. Although there is considerable variation in the measurement of SRH in these studies, some using noncomparative SRH measures (respondents rate their health as excellent, very good, good, fair, or poor) and others using comparative SRH measures (respondents rate their health as better, same, or worse compared to other people of their age), it has been shown that comparative and noncomparative measures of SRH do represent parallel assessments of subjective health [35]. 

Positive and negative states of health may be regarded as distinct elements of overall health, not merely mirror images of each other [36–39]. Hence, it is important to distinguish between health dimensions that are associated with a positive perception of health from those associated with a negative perception. Doing so would also help prioritize among various health dimensions; improving those that improve positive SRH as well as reduce negative SRH is likely to be more beneficial than improving those associated with only one or none of the two states. 

Thus, the current study aimed to provide an overall assessment of health, spanning 15 distinct health dimensions including overall SRH, of older adults in Singapore, and examine the association of positive and negative SRH with the other, more specific, health dimensions.

## 2. Materials and Methods

### 2.1. Social Isolation, Health and Lifestyles Survey (SIHLS) 2009

 SIHLS, a nationally representative survey of community-dwelling Singaporeans aged 60 years and above, was conducted by the Ministry of Community Development, Youth and Sports (MCYS), Singapore. MCYS provided a sample of 8,400 older adults, assuming a 60% response rate with a target sample size of 5,000, stratified by gender, ethnic group, and 5-year age groups based on the 2007 population distribution, from the national database of dwellings. Older adults aged ≥75 years, and Malays and Indians were oversampled by a factor of two to ensure sufficient numbers in these groups for analysis. Excluding 1,195 (14.2%) with invalid addresses, 5000 older adults were interviewed at their residence, with informed consent (response rate 69.4%). Proxy respondents (for 458 (9.2%) older adults unable to respond due to health reasons) were not asked questions on sleep, social networks outside the household, depressive symptoms, personal mastery, and perceived financial adequacy.

### 2.2. Fifteen Dimensions of Health

#### 2.2.1. SRH

SRH was based on the question “in general, how would you describe your state of health?” Responses were categorized as positive (“very healthy”, “healthier than average”), “average”, and negative (“somewhat unhealthy”, “very unhealthy”) SRH.

#### 2.2.2. Activities of Daily Living (ADLs)

 Older adults were classified as independent in ADLs if they reported no difficulty in any of the seven activities of bathing, dressing, eating, toileting, standing up and sitting down on bed/chair, walking (around the house), and going outside the house.

#### 2.2.3. Instrumental ADLs (IADLs)

Study participants were regarded as independent in IADLs (preparing meals, leaving the house to purchase medication, taking care of financial matters, using the phone, dusting/cleaning and other light house work, taking public transport, and taking medication as prescribed) if they either experienced no difficulty in any of these activities or did not perform them due to non-health/physical reasons.

#### 2.2.4. Mobility

Older adults were deemed to be independent in mobility if they had no difficulty in upper and lower extremity functions, based on Nagi's measures of physical performance (walk 200–300 metres, climb 10 steps without resting, stand for 2 hours, sit for 2 hours, stoop/bend knees, raise hands above the head, extend arms in front as if to shake hands, grasp with fingers or move fingers easily, and lift an object weighing 10 kg) [40].

#### 2.2.5. Hearing and Vision

Vision was assessed by asking “with your glasses or contact lenses, if you wear them, is your eyesight excellent, very good, good, fair, poor or loss of vision in both eyes” and hearing by “is your hearing excellent, very good, good, fair, poor or not able to hear in both ears? If you use hearing aids, please respond based on hearing ability when you wear them”. Those responding excellent, very good, or good to these questions were deemed to have no difficulty in vision and hearing, respectively.

#### 2.2.6. Major Physical Illness

Older adults were classified as being free from major physical illness if they answered no to a series of questions (Have you ever been diagnosed by a medical professional with …) relating to specific illnesses that included angina/myocardial infarction, other heart diseases, cancer (excluding skin cancer), diabetes, and cerebrovascular diseases.

#### 2.2.7. Pain

Pain was assessed through the question “overall in the last 30 days, how much of bodily aches or pains (in terms of intensity), did you have?” Those responding “none” or “mild” were categorized as being free from pain (other potential responses were “moderate”, “severe”, and “extreme/cannot function because of pain”).

#### 2.2.8. Sleep

Quality of sleep was assessed by asking “how often do you feel really rested when you wake up in the morning? Would you say most of the time, sometimes, or rarely or never?” Those responding “most of the time” were considered to have restful or restorative sleep.

#### 2.2.9. Biting/Chewing Ability

Older adults who were able to bite/chew most food items including *ikan bilis* (anchovies), shredded dry squid, mutton curry, dry mango, fresh carrots, *bak-kwa* (dried meat), bread with crust, *kang kong* (water spinach), *chicken satay*, or raw cucumber, were considered to have a “strong” biting/chewing ability. Those who could only eat thai rice, fried fish ball, wanton noodles, bananas, ripe papaya, hardboiled egg, or unable to chew any of the food items asked were classified as having “weak” biting/chewing ability.

#### 2.2.10. Depressive Symptoms

Depressive symptoms were assessed using the 11-item CES-D (Center for Epidemiologic studies for Depression) scale, the total score ranging from 0 to 22. Those with a score of <7 were classified as free of clinically relevant depressive symptoms [41].

#### 2.2.11. Personal Mastery

Five of the seven items from Pearlin's personal mastery scale were used to assess personal mastery. Responses for each item were scored on a 4-point agree-disagree format. Total score ranged from 0 to15, respondents with scores ≥25th percentile categorized as having “strong” personal mastery [42, 43].

#### 2.2.12. Participation in Social Activities

This was assessed through frequency (every day/every week/every month/less than once a month/not at all) of attending a residents/community development committee or neighbourhood event and of attending a place of worship. Involvement in one or both activities was considered as participation in social activities.

#### 2.2.13. Social Networks outside the Household

Lubben's revised social network scale was modified to assess social networks of the older adults outside the household. The scale consisted of 12 items (6 each for social networks with friends and with relatives outside of the household) assessing the size of network, frequency of contact, closeness, and perceived support from friends and relatives outside of the household [44]. Each item was scored on a 6-point scale, from 0 to 5. The sum of scores ranged from 0 to 60, 0 indicating the lowest and 60 the highest possible score. Those with a score ≥25th percentile were classified as having ‘strong' social networks outside the household.

#### 2.2.14. Perceived Financial Adequacy

Those responding “enough money, with some left over” or “just enough money” to the question “do you feel that you have adequate income to meet your expenses per month?" (other response options were “some difficulty to meet expenses” and “much difficulty to meet expenses”) were considered to perceive their finances as adequate.

### 2.3. Statistical Analysis

Prevalence of SRH and “health” in each of the other 14 dimensions was estimated, overall and by age, gender, and ethnicity. Bivariable and multivariable generalized logit models were used to assess the relative contribution of each of the 14 health dimensions to positive and negative SRH, compared to average SRH. The outcome variable (positive, average, and negative SRH) was treated as a nominal rather than an ordinal variable as the proportional odds assumption that *β*_*k*_ = *β* for all *k*, where *k* indexes both the logits, was not met. The multivariable models adjusted for sociodemographic variables (age, gender, ethnicity, highest educational level completed (none, primary, secondary, vocational/junior college/polytechnic, university, and above), marital status (married, widowed, separated/divorced, never married), and housing type as a proxy for socioeconomic status (1-2 room public, 3 room public, 4-5 room public/condominium/bungalow/shop house)). The analysis was restricted to Chinese, Malays, and Indians, the three largest ethnic groups in Singapore. Respondents belonging to the “Other” ethnic group (1.2%) were excluded as their numbers were too low for meaningful interpretation. The analyses, conducted using SAS, version 9.2 (SAS Institute, Inc., Cary, NC), were weighted to adjust for nonresponse and oversampling of Malays and Indians, and those 75 years and older. Approval for analysis was obtained from IRB boards at National University of Singapore and Duke University Health System.

## 3. Results

 More than four-fifth older Singaporeans in the overall sample rated themselves to be healthy (i.e., have average or positive SRH). The prevalence of positive SRH was 40%, being higher among women, Chinese, and those aged 60–74 years. Older age group (≥75 years) had worse health in most dimensions compared to those aged 60–74 years. This difference was greatest for independence in mobility (*P* < 0.001, chi-square test), especially among women, with only 7–14% women aged 75 years and above being independent. Indians had the highest prevalence of physical illness among the ethnic groups. Men were more likely to report freedom from pain than women. They were also less likely to report clinically relevant depressive symptoms than women in the same age group. Prevalence of restful sleep and perceived financial adequacy was higher among women than men and among Chinese than Malays and Indians (Table 1).

In unadjusted analysis, health in most of the dimensions was associated with increased odds of positive SRH (except independence in mobility, restful sleep, and strong social networks) and decreased odds of negative SRH (except restful sleep), relative to average SRH. In multivariable analysis, the strongest relationships with SRH were seen for mobility, vision, major physical illness, pain, personal mastery, depressive symptoms, and perceived financial adequacy—health in these dimensions was associated with significantly increased odds of positive SRH and decreased odds of negative SRH. Restful sleep, strong biting/chewing ability, and hearing were associated only with positive SRH. Conversely, independence in IADLs was inversely associated with only negative SRH (Table 2).

Age was associated with both SRH states in the unadjusted analysis; the relationship, however, disappeared in the adjusted model. Women (versus men) were more likely to have positive SRH. Malays and Indians (versus the Chinese) had lower odds of both positive and negative SRH. Higher education increased the odds of positive SRH and reduced negative SRH. Living in smaller housing was associated with negative SRH in the unadjusted analysis, but with positive SRH in the adjusted analysis (Table 2).

## 4. Discussion

The study presents a holistic picture of health status of older Singaporeans. Overall, 85.5% rated their health to be average or higher. Positive and negative SRH were associated with most of the dimensions studied; the strongest associations were seen with mobility, hearing, vision, major physical illness, pain, personal mastery, depressive symptoms, and perceived financial adequacy. 

As expected, older adults aged ≥75 years reported worse health in all dimensions compared to those aged 60–74 years. Women were more likely to report positive SRH than men, even though they were worse off than men in most of the other health dimensions. While most studies report a contrary finding, that is, a female disadvantage in SRH [22, 45], findings similar to ours have also been reported [46]. Better SRH among women could reflect their lower expectation of health than men or indicate a variation in the meaning of SRH between genders. Higher education increased positive SRH and reduced negative SRH, as reported previously [30]. Malays and Indians had significantly lower odds of both positive and negative SRH than the Chinese, suggesting that they were more likely to choose midpoint values and rate their health as average compared to Chinese, possibly due to differences in perception of SRH between ethnic groups.

 Freedom from pain had the strongest association with positive SRH. Very few studies have reported this association among older adults [32, 33]. A greater emphasis on assessment and treatment of pain among older adults by health practitioners may, thus, be beneficial in improving SRH. Our findings that pain prevalence is higher among women and increases with age, are consistent with previous studies on pain epidemiology [47–50]. 

Presence of physical illness was a strong correlate of negative SRH, consistent with previous studies, though specific diseases are considered and operationalization of SRH is varied [22, 23, 26, 27]. Indians had the highest prevalence of major physical illness, corroborating previous studies that have noted a higher mortality from ischemic heart diseases among Indians compared to Chinese and Malays [51]. Reducing the prevalence and incidence of these illnesses could thus improve SRH. We also find independence in functional activities, especially IADLs and mobility, to be associated with lower odds of negative SRH, suggesting that SRH can be improved if independence in these activities can be achieved. 

 The association of absence of sensory impairments with positive SRH, independent of functional deficits, has been reported before [23, 25]. Though some deterioration in vision and hearing with age is almost universal, their impact on overall health can be reduced through prevention (e.g., strict glycaemic control for diabetes) and appropriate management (e.g., use of eyeglasses for improving visual acuity, surgical interventions for cataract and hearing aids for hearing impairment). 

We find that about 45% of older Singaporeans have restful sleep. Lack of restful sleep is known to be associated with falls, attention deficits, decline in short-term memory, performance levels, and cognition among the older adults [52]. Restful sleep contributed to positive SRH, though its absence was not associated with negative SRH. Managing nonrestful sleep through pharmacological and nonpharmacological measures may help improve the subjective health status of older adults. The prevalence of nonrestful sleep was found to increase with age, possibly a result of factors such as medical and psychiatric illnesses, medication use, circadian rhythm changes, sleep disordered breathing, and REM disorders [52]. Lower prevalence of restful sleep among Malays and Indians may be related to their higher body mass and hypertension prevalence [53], while a higher prevalence among men may be due to obstructive sleep apnoea being more common among them than women [54].

 Oral health, considered to be an important component of active aging by the WHO [55], was found to contribute to positive SRH. A study from Japan also noted good chewing ability to be related to excellent/good SRH [23]. Problems in mastication not only result in poor or less diverse diet, affecting nutritional intake, but also can discourage older adults from enjoying meals with family and friends, interfering in their social relations, and possibly reducing their SRH [23, 31, 56]. 

Corroborating previous studies [23, 26, 27], absence of depressive symptoms, and perceived financial adequacy were associated with both SRH states [27, 57]. Another salient finding of the present analysis, noted by only a few studies [30, 58], was the relationship between personal mastery and SRH. Strong personal mastery promotes well-being through participation in health-promoting activities, maintains positive psychological states, and acts as a buffer against external stressors [58]. Conversely, low self-esteem/control makes one more prone to stress, contributing to negative SRH. 

 Strengths of the study include its large and nationally representative sample, allowing generalizability to the older population of Singapore, and examination of the association of SRH with health dimensions such as pain, sleep, chewing ability, and personal mastery, which have been comparatively less studied than other health dimensions such as physical illness and functional limitations. We did not consider factors such as physical activity, smoking, and body mass status, often associated with SRH, as health dimensions in our analysis since it was felt that these are risk factors for poor health, rather than separate health dimensions. 

Limitations of the study include its cross-sectional design, limiting us in assigning concrete causal associations between SRH and other health dimensions. Further, any comparison of SRH with other countries is difficult due to differences in method of capturing SRH status. The various health dimensions are self-reported, thus subjected to reporting bias and differences in interpretation between various population subgroups. Nevertheless, this study makes an important contribution to the literature by presenting a comprehensive picture of the health status of older adults in Singapore and supports a multidimensional understanding of health status.

## 5. Conclusion

The findings provide evidence to policy makers interested in improving the overall health of older adults. We find that while some decline in overall health is a consequence of aging, vast majority of older Singaporeans report themselves as healthy overall and in a wide range of health dimensions which is encouraging. Some of these health dimensions associated with both positive and negative SRH are potentially modifiable and thus provide avenue for further improvements in SRH.

## Figures and Tables

**Table 1:** Prevalence of self-rated health and other health dimensions.

Variables	Total (*n* = 4941) % (*n*)	Men (*n* = 2229)						Women (*n* = 2712)					
		60–74 years (*n* = 1355)			≥75 years (*n* = 874)			6–74 years (*n* = 1508)			≥75 years (*n* = 1204)		
		Chinese (*n* = 966) % (*n*)	Malay (*n* = 244) % (*n*)	Indian (*n* = 145) % (*n*)	Chinese (*n* = 567) % (*n*)	Malay (*n* = 172) % (*n*)	Indian (*n* = 135) % (*n*)	Chinese (*n* = 1102) % (*n*)	Malay (*n* = 262) % (*n*)	Indian (*n* = 144) % (*n*)	Chinese (*n* = 941) % (*n*)	Malay (*n* = 176) % (*n*)	Indian (*n* = 87) % (*n*)
Self-rated health													
*Very healthy*	9.5 (416)	5.0 (47)	8.3 (19)	7.7 (11)	5.1 (29)	1.3 (2)	2.1 (3)	16.1 (175)	4.5 (11)	7.2 (10)	11.0 (104)	0.5 (1)	4.7 (4)
*Healthier than average*	30.7 (1385)	24.4 (234)	13.8 (33)	12.7 (18)	12.6 (71)	7.1 (13)	3.4 (5)	43.4 (478)	43.0 (114)	37.1 (53)	31.7 (299)	24.3 (41)	29.6 (26)
*Of average health*	45.3 (2326)	55.8 (535)	70.9 (173)	73.6 (106)	55.5 (315)	71.9 (123)	75.2 (103)	31.5 (345)	38.6 (101)	41.1 (60)	37.2 (349)	40.6 (72)	50.3 (44)
*Somewhat unhealthy*	12.1 (674)	12.9 (131)	5.1 (15)	4.9 (8)	22.3 (127)	17.7 (30)	18.0 (23)	7.9 (91)	8.5 (24)	11.2 (16)	16.4 (154)	24.4 (44)	12.3 (11)
*Very unhealthy*	2.3 (137)	1.7 (17)	1.8 (4)	1.1 (2)	4.2 (24)	2.0 (4)	1.3 (1)	1.2 (13)	5.3 (12)	3.5 (5)	3.7 (35)	10.3 (18)	3.1 (2)
Independent in activities of daily living	90.0 (4252)	95.8 (925)	96.9 (236)	97.7 (141)	83.7 (473)	88.4 (151)	86.0 (118)	96.1 (1055)	88.9 (231)	85.5 (122)	69.2 (654)	55.3 (96)	56.2 (50)
Independent in instrumental activities of daily living	86.7 (4034)	95.7 (922)	97.8 (239)	98.3 (142)	81.7 (461)	84.4 (144)	85.3 (116)	93.0 (1017)	86.1 (223)	80.3 (114)	57.2 (542)	41.9 (72)	47.2 (42)
Independent in mobility	54.2 (2352)	76.9 (731)	86.7 (209)	86.3 (124)	41.9 (235)	66.0 (112)	59.0 (80)	52.5 (555)	44.8 (110)	26.7 (37)	13.9 (131)	13.0 (22)	6.6 (6)
No difficulty in hearing	85.2 (4035)	87.5 (835)	91.6 (220)	88.4 (127)	68.0 (383)	72.8 (124)	76.3 (106)	91.4 (1004)	91.3 (239)	93.7 (135)	74.5 (702)	56.6 (97)	72.0 (63)
No difficulty in vision	78.9 (3764)	81.0 (779)	88.7 (215)	88.2 (126)	67.3 (380)	66.9 (114)	68.9 (95)	83.1 (913)	79.7 (208)	84.1 (121)	70.3 (663)	54.4 (94)	63.9 (56)
No major physical illness	67.8 (3177)	69.7 (665)	65.2 (153)	49.5 (71)	59.4 (336)	59.7 (103)	41.4 (55)	74.7 (810)	61.9 (159)	53.8 (76)	64.0 (601)	61.3 (110)	43.7 (38)
Free from pain	88.5 (4276)	95.2 (916)	93.1 (226)	95.3 (138)	91.2 (517)	87.6 (151)	88.6 (121)	87.5 (959)	84.1 (220)	75.5 (108)	79.1 (746)	70.0 (122)	60.7 (52)
Restful sleep (*n* = 4489)^a^	44.6 (1967)	51.8 (492)	47.8 (115)	30.8 (44)	50.0 (239)	36.8 (57)	28.9 (36)	39.2 (412)	66.5 (159)	35.9 (50)	34.5 (264)	60.1 (70)	43.4 (29)
Strong biting/chewing ability	86.6 (4059)	91.2 (875)	88.2 (213)	91.9 (132)	72.4 (408)	74.9 (127)	72.6 (98)	94.7 (1036)	87.6 (229)	92.1 (132)	66.9 (632)	69.7 (122)	62.2 (55)
Free from depressive symptoms (*n* = 4489)^a^	85.0 (3758)	87.6 (822)	93.8 (225)	85.0 (121)	84.0 (400)	94.6 (142)	86.3 (108)	86.5 (928)	73.9 (181)	75.0 (102)	78.0 (596)	72.2 (85)	72.4 (48)
Strong personal mastery (*n* = 4489)^a^	75.4 (3314)	74.7 (701)	92.4 (219)	79.1 (112)	69.4 (331)	86.8 (131)	74.5 (92)	79.8 (851)	74.3 (180)	67.5 (91)	65.3 (498)	56.5 (66)	62.8 (42)
Participation in social activities	71.8 (3438)	65.4 (628)	95.0 (232)	91.5 (132)	46.4 (262)	86.1 (148)	86.0 (118)	84.5 (930)	63.9 (165)	85.2 (122)	64.1 (605)	21.4 (37)	66.9 (59)
Strong social networks (*n* = 4489)^a^	77.9 (3401)	83.1 (783)	81.3 (190)	72.1 (103)	78.3 (373)	80.9 (121)	60.6 (76)	79.1 (850)	77.4 (191)	67.3 (92)	64.9 (494)	66.6 (79)	73.4 (49)
Perceived financial adequacy (*n* = 4489)^a^	82.6 (3587)	80.5 (754)	57.6 (134)	66.1 (92)	77.8 (371)	45.7 (69)	58.8 (74)	89.9 (968)	77.5 (190)	81.4 (112)	88.1 (672)	79.1 (93)	87.1 (58)

^a^were not asked to proxy respondents. Thus the “*n*” in each cell is lower.

**Table 2:** Unadjusted and adjusted odd ratios for positive and negative self-rated health among older adults.

Variables	Unadjusted odds ratios (95% CI) (*n* = 4489)^a^		Adjusted odds ratios (95% CI) *N* = 4211	
	Positive^b^ versus average self-rated health	Negative^c^ versus average self-rated health	Positive^b^ versus average self-rated health	Negative^c^ versus average self-rated health
Sociodemographic				
*Age * 65–74 versus 60–64	1.0 (0.9–1.1)	1.4 (1.1–1.8)	1.2 (1.0–1.4)	0.9 (0.7–1.2)
75 and above versus 60–64	0.7 (0.6–0.9)	1.9 (1.5–2.4)	1.1 (0.9–1.4)	0.9 (0.6–1.3)
*Gender * Females versus Males	3.6 (3.2–4.1)	1.2 (1.0–1.5)	4.7 (3.9–5.6)	0.6 (0.4–0.8)
*Ethnicity * Malay versus Chinese	0.6 (0.5–0.8)	0.7 (0.5–0.9)	0.6 (0.5–0.8)	0.5 (0.3–0.8)
Indians versus Chinese	0.5 (0.4–0.6)	0.6 (0.4–0.8)	0.6 (0.5–0.9)	0.5 (0.3–0.7)
*Educational status * Primary versus none	0.8 (0.7–1.0)	0.9 (0.7–1.1)	1.1 (0.9–1.3)	1.2 (0.9–1.6)
Secondary versus none	0.9 (0.7–1.0)	0.6 (0.5–0.8)	1.2 (1.0–1.5)	1.0 (0.7–1.4)
Vocational/junior college/polytechnic/university and above versus none	1.0 (0.8–1.2)	0.3 (0.2–0.5)	1.5 (1.1–2.0)	0.5 (0.3–0.9)
*Housing type * 1-2 room public housing versus 4-5 room	1.0 (0.7–1.2)	1.6 (1.1–2.2)	1.5 (1.1–2.1)	1.2 (0.8–1.9)
public/private3 room public housing versus 4-5 room public/private	1.0 (0.9–1.2)	1.2 (1.0–1.5)	1.1 (1.0–1.4)	1.2 (0.9–1.5)
*Marital status * Widowed versus married	1.5 (1.3–1.7)	1.2 (1.0–1.5)	1.2 (1.0–1.4)	0.7 (0.5–1.0)
Separated/divorced versus married	2.2 (1.5–3.2)	2.5 (1.5–4.1)	1.9 (1.2–2.9)	2.2 (1.2–4.0)
Never married versus married	1.7 (1.3–2.3)	1.8 (1.2–2.6)	1.2 (0.8–1.7)	1.5 (0.9–2.5)

Health dimensions				

Independent in activities of daily living	2.1 (1.5–2.9)	0.2 (0.1–0.2)	1.4 (0.8–2.2)	0.6 (0.4–1.0)
Independent in instrumental activities of daily living	1.5 (1.1–1.9)	0.2 (0.1–0.2)	1.1 (0.8–1.7)	0.5 (0.3–0.7)
Independent in mobility	1.1 (0.9–1.2)	0.2 (0.2–0.3)	1.3 (1.1–1.6)	0.3 (0.2–0.4)
No difficulty in hearing	2.8 (2.2–3.6)	0.4 (0.3–0.5)	1.5 (1.2–2.1)	0.8 (0.6–1.0)
No difficulty in vision	2.9 (2.4–3.5)	0.3 (0.3–0.4)	2.7 (2.1–3.4)	0.5 (0.4–0.6)
No major physical illness	2.1 (1.8–2.4)	0.3 (0.2–0.4)	1.9 (1.6–2.3)	0.4 (0.3–0.4)
Free from pain	3.2 (2.4–4.2)	0.3 (0.2–0.3)	3.3 (2.4–4.6)	0.5 (0.4–0.7)
Restful sleep	1.2 (1.0–1.3)	1.1 (0.9–1.3)	1.4 (1.2–1.6)	1.2 (0.9–1.5)
Strong biting/chewing ability	2.1 (1.7–2.6)	0.6 (0.4–0.7)	1.4 (1.1–1.8)	1.4 (1.0–2.0)
Free from depressive symptoms	2.2 (1.8–2.7)	0.3 (0.3–0.4)	1.6 (1.3–2.1)	0.7 (0.5–0.9)
Strong personal mastery	2.0 (1.7–2.3)	0.4 (0.4–0.5)	1.8 (1.5–2.2)	0.7 (0.5–0.9)
Participation in social activities	1.4 (1.2–1.7)	0.5 (0.4–0.7)	1.0 (0.9–1.3)	0.9 (0.7–1.2)
Strong social networks	1.1 (0.9–1.2)	0.7 (0.6–0.8)	1.0 ( 0.8–1.2)	1.0 (0.8–1.3)
Perceived financial adequacy	3.7 (3.0–4.5)	0.5 (0.4–0.6)	2.2 (1.8–2.8)	0.6 (0.4–0.8)

^a^ *n* for each odds ratio may vary due to variable number of missing values.

^b^Positive self-rated health includes those who respond “very healthy” or “healthier than average”.

^c^Negative self-rated health includes those who respond “somewhat unhealthy” or “very unhealthy”.
